# Darovasertib, a novel treatment for metastatic uveal melanoma

**DOI:** 10.3389/fphar.2023.1232787

**Published:** 2023-07-28

**Authors:** Lei Cao, Shuzhen Chen, Rainie Sun, Charles R. Ashby, Liuya Wei, Zoufang Huang, Zhe-Sheng Chen

**Affiliations:** ^1^ Ernest Mario School of Pharmacy, Rutgers, The State University of New Jersey, New Brunswick, NJ, United States; ^2^ School of Pharmacy, Weifang Medical University, Weifang, China; ^3^ Department of Pharmaceutical Sciences, College of Pharmacy and Health Sciences, St. John’s University, New York, NY, United States; ^4^ Stuyvesant High School, New York, NY, United States; ^5^ Ganzhou Key Laboratory of Hematology, Department of Hematology, The First Affiliated Hospital of Gannan Medical University, Ganzhou, Jiangxi, China

**Keywords:** metastatic uveal melanoma, darovasertib, PKC inhibitors, GNAQ/11, binimetinib, crizotinib

## Abstract

The FDA granted orphan drug designation to darovasertib, a first-in-class oral, small molecular inhibitor of protein kinase C (PKC), for the treatment of uveal melanoma, on 2 May 2022. Primary uveal melanoma has a high risk of progressing to metastatic uveal melanoma, with a poor prognosis. The activation of the PKC and mitogen-activated protein kinase pathways play an essential role in the pathogenesis of uveal melanoma, and mutations in the G protein subunit alpha q (GNAQ), and G protein subunit alpha11 (GNA11) genes are considered early events in the development of uveal melanoma. Compared to other PKC inhibitors, such as sotrastaurin and enzastaurin, darovasertib is significantly more potent in inhibiting conventional (α, β) and novel (δ, ϵ, η, θ) PKC proteins and has a better tolerability and safety profile. Current Phase I/II clinical trials indicated that darovasertib, combined with the Mitogen-activated protein kinase/Extracellular (MEK) inhibitors, binimetinib or crizotinib, produced a synergistic effect of uveal melanoma. In this article, we summarize the development of drugs for treating uveal melanomas and discuss problems associated with current treatments. We also discuss the mechanism of action, pharmacokinetic profile, adverse effects, and clinical trial for darovasertib, and future research directions for treating uveal melanoma.

## Introduction

Among all the primary intraocular cancers, uveal melanoma is one of the most common types in adults. According to data from the American Cancer Society, there are 7,095 new cases of uveal melanoma diagnosed yearly, with a mean age-adjusted incidence of 4.3 per million people (Barker and Salama, 2018). For local uveal melanoma, radiation therapies, such as proton therapy and plaque brachytherapy, are standard treatments (Barker and Salama, 2018). However, approximately 40%–50% of uveal melanoma patients eventually develop metastatic disease, most commonly in the liver (Kaliki and Shields, 2017). Patients diagnosed with metastatic disease usually have a poor prognosis, and the median overall survival is only 10 months (Barker and Salama, 2018). The mortality rate is estimated to be 31% and 49%, for 5 and 25 years, respectively, from the time of primary tumor diagnosis, due to the lack of effective therapies once the disease has progressed to the metastatic phase (Kaliki and Shields, 2017). As shown in Table 1, many targeted therapies and immunotherapies are being evaluated in clinical trials for metastatic uveal melanoma. However, current targeted therapy does not produce an optimal therapeutic outcome for metastatic uveal melanoma (Barker and Salama, 2018).

**TABLE 1:** Current immunotherapies and targeted therapies undergoing clinical trials for metastatic uveal melanoma.

	Drug category	Therapeutic targets	Generic name	Indication	Current status
1	CTLA-4 inhibitor	T-cell receptors	ipilimumab	metastatic uveal melanoma	Phase II Clinical trial
2	PD-1 inhibitor	T-cell receptors	nivolumab	metastatic uveal melanoma	Phase II Clinical Trial
3	PD-1 inhibitor	T-cell receptors	Pembrolizumab	metastatic uveal melanoma	Phase II Clinical trial
4	gp100- inhbitor	TCR/Anti-CD3 bispecific fusion protein	Tebentafusp	metastatic uveal melanoma	Phase II Clinical Trial
5	MEK inhibitors	G protein coupled downstream signaling cascade pathway	Selumetinib + dacarbazine	metastatic uveal melanoma	Phase III
6			Trametinib	metastatic uveal melanoma	Phase II
7			Binimetinib + darovasertib	metastatic uveal melanoma	Phase 1b/II
8			Cabozantinib	metastatic uveal melanoma	Phase II
9	PI3K/Akt/mTOR inhibitors	Inhibit the PI3K/Akt pathway	Everolimus combined with pasireotide	metastatic uveal melanoma	Phase II
10	VEGF inhibitor+ chemotherapy	Inhibits vascular endothelial growth factor-related pathway	Bevacizumab+ temozolomide	metastatic uveal melanoma	Phase II
11	VEGF inhibitor		Cabozantinib	metastatic uveal melanoma	Phase II
12	HDAC inhibitors	block the activity of histone deacetylase enzymes	Vorinostat	metastatic uveal melanoma	Phase II
13	C-Met inhibitor	inhibit c-MET and HGFR (a hepatocyte growth factor receptor)	Crizotinib	metastatic uveal melanoma	Phase II

### Tebentafusp produces a low therapeutic response

Tebentafusp was approved by the United States Food and Drug Administration (FDA) on 25 January 2022, as the first systemic therapy for metastatic uveal melanoma (Center for Drug Evaluation and and Research, 2022). Tebentafusp is an immune-mobilizing, monoclonal T-cell receptor that utilizes an HLA-A*02:01-restricted T-cell receptor with high specificity for the gp100 peptide, which is a melanocyte lineage-specific antigen expressed by lymphocytes that infiltrate tumors, and gp100 peptide expression is significantly positively correlated with metastatic melanoma tumor progression (Middleton et al., 2020). However, a Phase II clinical trial indicated that the median survival time for patients is 6–12 months, regardless of the treatment (Nathan et al., 2021). The response rate was 9% in the tebentafusp group, compared to 5% in the control group and the duration of the response was similar for the control and tebebtafusp groups (9.9 vs 9.7 months, respectively) (Nathan et al., 2021). It is important to note that in the clinical trial for tebentafusp, patients had to have the genotype, HLA-A*02:01, which is present in 50% of the population (Cole et al., 2020).

### Immune checkpoint inhibitors are not efficacious in patients with metastatic uveal melanoma

Immune checkpoint inhibitors are efficacious in patients with a high tumor burden (Tang et al., 2018), including patients with cutaneous melanoma, which has one of the highest tumor burdens of any solid tumor (Leiter et al., 2004). In contrast, uveal melanoma has a low tumor burden, and current single-therapy immune checkpoint inhibitors approved for treating cutaneous melanoma have been reported to lack significant efficacy (Algazi et al., 2016). Ipilimumab did not produce a significant therapeutic response in patients with uveal melanoma, and the median overall survival was 6.8 months (Algazi et al., 2016). Furthermore, patients did not show a response to tremelimumab, with a median overall survival of 12.8 months (Algazi et al., 2016). In patients diagnosed with uveal melanoma, drugs that inhibit the programmed death-1 pathway (PD-1) (e.g., nivolumab and pembrolizumab) produced an overall response rate of 3.6% and a median overall survival of 7.6 months (Joshua et al., 2015). Although the combinations of specific immune checkpoint inhibitors are more efficacious than monotherapy, the combination of nivolumab and ipilimumab only produced a 15%–18% overall response rate, indicating that the therapeutic outcome is suboptimal (Piperno-Neumann et al., 2020). Of 64 patients who participated in the trials, the 1- year overall survival rate was 56%. (Piperno-Neumann et al., 2020).

### Targeted therapies for uveal melanoma

Because of the resistance to current chemotherapy and the lack of efficacy of the immune checkpoint inhibitors, more novel therapies, specifically compared to chemotherapies, that target uveal melanoma at different signaling pathways are urgently needed. Novel therapies for metastatic uveal melanoma include drugs targeting the 1) MAPK pathway, such as the selective MEK1/2 inhibitor, selumetinib; 2) PKC pathway, such as AEB071 (Pelster et al., 2021) and IDE196 (i.e., darovasertib (Ideayabio, 2022a)); 3) phosphoinositide-3-kinase (PI3K) and insulin-like growth factor-1(IGF)-1/insulin-like growth factor type 1 receptor (IGF-1R) pathways, e.g., pasireotide and 4) Hippo-Yes-associated protein 1(YAP) pathway (Martin et al., 2013). Novel treatments targeting late prognostic mutations in the Gα pathway and epigenetic regulation are being extensively investigated (Chen et al., 2014). This category includes compounds that inhibit histone deacetylase (HDAC) (Landreville et al., 2012), Histone-lysine N-methyltransferase (EZH2) (Chen et al., 2014)^,^ and poly (ADP-ribose) polymerase (PARP) (Sagoo et al., 2014). Other epigenetic regulatory proteins, such as the bromodomain and extra terminal (BET; Harbour et al., 2013) protein families, bromodomain-containing protein 4 (BRD4) (Chokhachi Baradaran et al., 2020)^,^ and barrier-to-autointegration factor (BAF) (Kuznetsoff et al., 2021) (mammalian SWItch/Sucrose Non-Fermentable (SWI/SNF) or Brahma-associated factor complexes, may be suitable targets for novel medications that can treat metastatic uveal melanoma.

Among all the aforementioned drugs, the PKC inhibitors, like sotrastaurin and darovasertib, are the most efficacious and safe treatments for uveal melanoma. Darovasertib is a first-in-class oral, small molecule inhibitor of protein kinase C that received approval as an orphan drug by the U.S. FDA on 2 May 2022, for treating uveal melanoma (Pharmabiz, 2022).

## Pathophysiology and pharmacogenetics

### GNA11 and GNAQ mutations play an important role in activating the PKC pathway

Activating mutations in the genes coding for G protein subunit alpha q (GNAQ) or G protein subunit alpha 11 (GNA11) are present in approximately 90% of uveal melanoma patients, and the GNA11 mutation rate is significantly dependent on PKC activity (Shoushtari and Carvajal, 2014). Thus, GNAQ and GNA11 are essential biomarkers for uveal melanoma in diagnostic panels. (Shoushtari and Carvajal, 2014).

**FIGURE 1:**
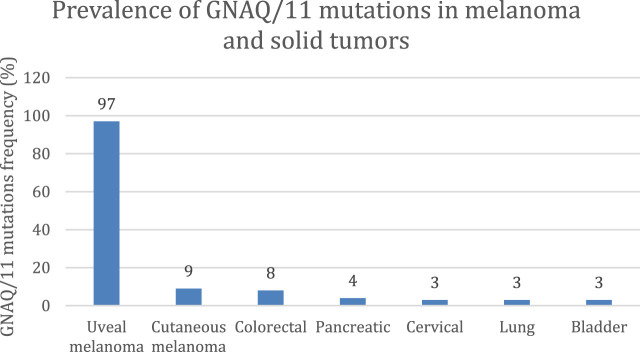
Prevalence of GNAQ/11 mutations in solid tumors (Shoushtari and Carvajal, 2014). At least 95% of uveal melanoma tumors have mutations in the genes coding for the proteins, GNAQ and/or GNA11. This mutation is also expressed in other solid tumors but at a lower frequency (<10%), including cutaneous melanoma, colorectal cancer, and pancreatic cancer.

**FIGURE 2:**
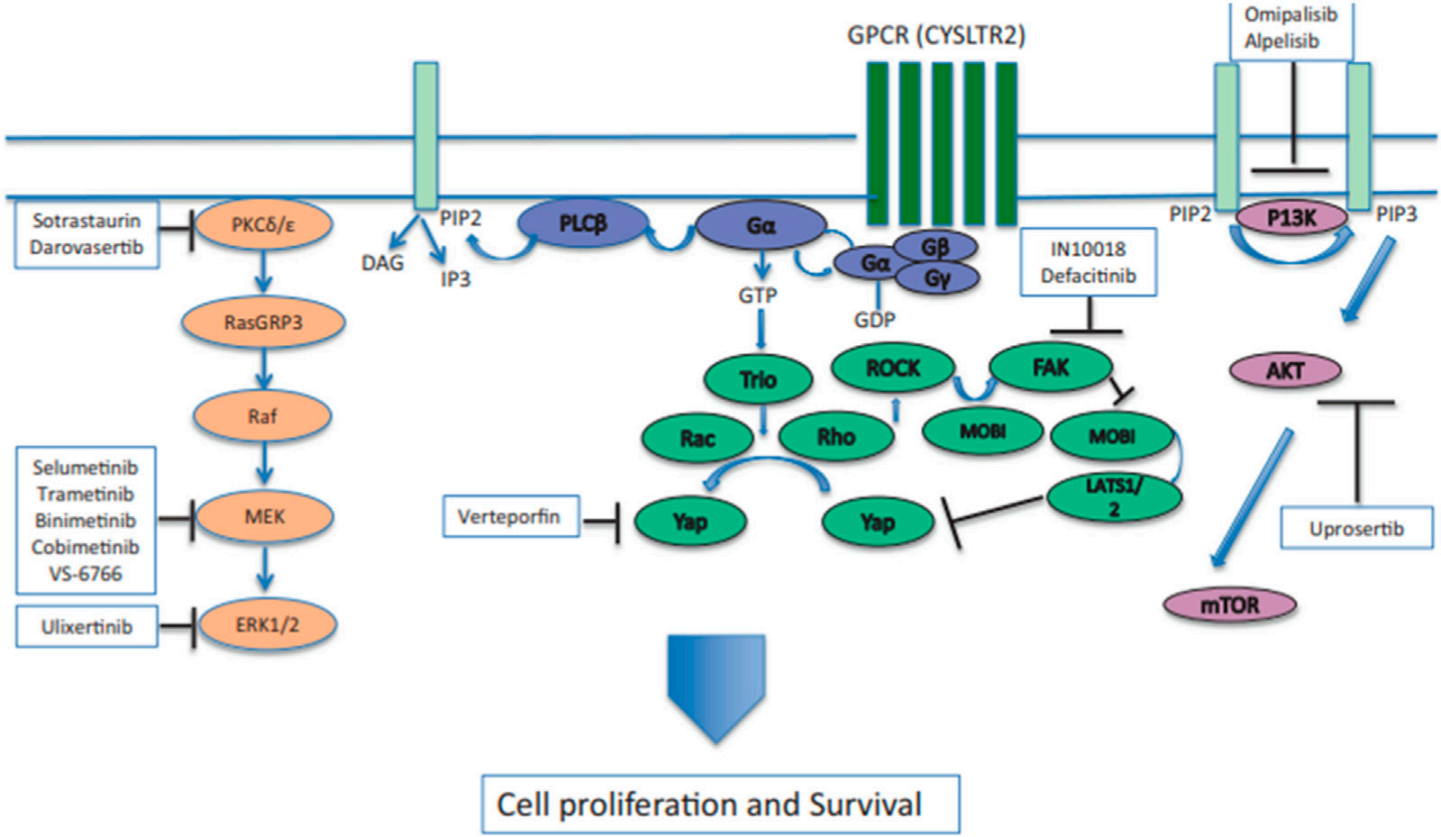
GNAQ or GNA11 mutations cause the constitutive activation of Gα, which activates signaling by activating phospholipase C (PLCβ) and protein kinase C (PKC). The activation of the RAS-dependent RAF pathway leads to the formation of the RAS-GTP–Raf-1 complex, inducing the activation of the downstream ERK1/2 pathway and the mitogen-activated protein kinase (MAPK) pathway. PKC inhibitors, such as sotrastuarin and darovasertib, inhibit the conventional PKC isoform, PKCβ, PKC, and the novel PKC isoforms, PKC δ and PKCε, thus suppressing the downstream signaling cascade, which decreases tumor cell proliferation and survival (Wei et al., 2022). Reproduced with permission from ref. (Wei et al., 2022). Copyright 2022, Springer Nature.

**FIGURE 3:**
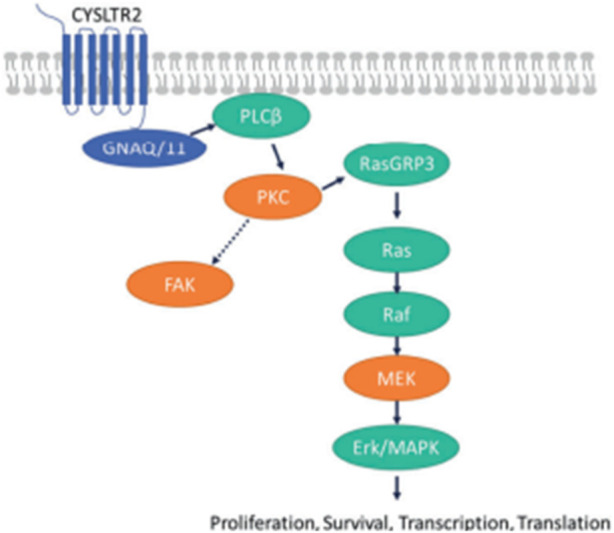
(Lietman and McKean, 2022). GNAQ/11 mutations are expressed at a higher level when the PKC pathway is activated. GNAQ and GNA11 mutations activate downstream signaling by upregulating the expression of the PKCβ isoform, activating the PKC pathway. The protein, RAS guanyl-releasing protein 3 (RasGRP3), is phosphorylated *in vitro* by PKC-θ and PKC-β2. In the RAS/RAF pathway, the activation of the PKC pathway activates the RAS-dependent Raf-1 protein kinase. This induces the formation of RAS-GTP–Raf-1 complexes, the complex that activates the ERK/MAPK signaling cascade, the key pathway in uveal melanoma and other solid tumors. The proteins, PKC, MEK and focal adhesion kinase (FAK), have been hypothesized to be key targets for novel medications in patients with metastatic uveal melanoma (Park et al., 2022). Reproduced with permission from ref. 75. Copyright 2022, Springer Nature.

As shown in Figure 1, GNAQ and GNA11 mutations are present in 55% and 50%, respectively, of primary uveal melanoma patients (Shoushtari and Carvajal, 2014). The amino acid mutations, Q209 P/L, R183Q or G48/V^23^, primarily occur in GNAQ, whereas the amino acids mutations, Q209L (94%), R183C (3%) or R166H (3%), primarily occur in GNA11 (Piperno-Neumann et al., 2014). These amino acid mutations cause the constitutive activation of the G protein and GTPase activity in GNAQ and GNA11 (Piperno-Neumann et al., 2014). (Seedor et al., 2021) As shown in Figure 2, the activation of Gαq and Gα11 subunits induces the activation of other G protein-coupled pathways, including PKC and MAPK, and PI3K.GNAQ and GNA11 activate the heterotrimeric G protein α-subunits that subsequently activate the enzyme, phospholipase C, which increases diacylglycerol (DAG) levels and recruits and activates conventional (α, β) and novel PKC (nPKC, δ, ϵ, η, θ) proteins (Seedor et al., 2021). As shown in Figure 3, GNAQ/11 mutations are expressed at a higher level when the PKC pathway is activated. The activation of the PKC pathway activates the Rat sarcoma virus (RAS)-dependent Rapidly Accelerated Fibrosarcoma (RAF)-1 protein kinase (Lietman and McKean, 2022). This induces the formation of RAS-GTP–Raf-1 complexes, which activates the ERK/MAPK signaling cascade, the key pathway in uveal melanoma and other solid tumors (Solus and Kraft, 2013). GNAQ and GNA11 mutations activate downstream signaling by upregulating the expression of the PKCβ isoform, activating the PKC pathway (Silva-Rodríguez et al., 2022). Thus, novel PKC inhibitors, such as sotrastaurin and enzastaurin, are being developed to target GNAQ and GNA11.

### The Interaction of the PKC and MAPK pathways

The PKC and MAPK pathways can significantly interact with one another to regulate certain cellular functions (Chen et al., 2017). For example, the MAPK pathway is activated by PKC and phospholipase β (PLCβ) (Cameron et al., 2009). Although PLCβ is ubiquitously expressed and plays a critical role in inflammation and cell signaling, research has led to the subsequent development of PKC inhibitors (Carracedo et al., 2014).

### The inhibition of PKC subtypes inhibits uveal melanoma proliferation

**FIGURE 4:**
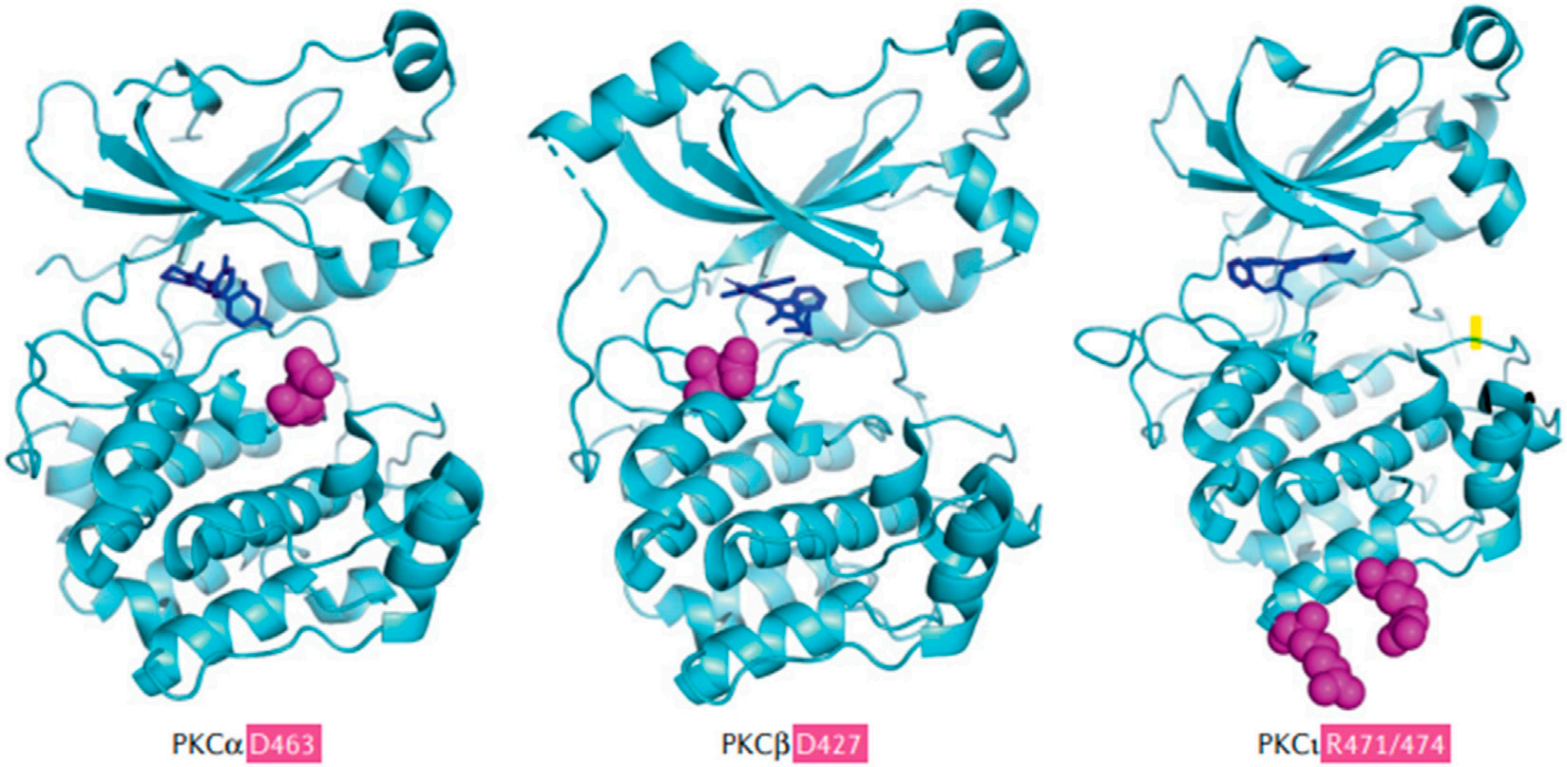
(Parker et al., 2021). The common PKC isoforms involved in mediating metastatic uveal melanoma (Cole et al., 2020). Sites of penetrant mutations (D463, D427, and R471/474) in the kinase domains of PKCα and PKCβ are highlighted as purple balls surrounded by the solved kinase domain structures (blue), alongside the hotspot and infrequent kinase domain mutations in the PKCι. The D463H mutation is a deactivating mutation, which can allow conformational change and priming for phosphorylation, producing the loss of catalytic activity and a decrease in the half-life of PKCα. D427N is an activating mutation in PKCβ and this decreases the probability of the binding of autoinhibitory pseudosubstrate short sequence at the N- terminus of PKCβ, which normally inhibits the conformational changes and activation of PKCβ. D427N increases the catalytic activity of mutated PKCβ and this facilitates tumor growth. PKCβ is the major PKC isoform expressed by colon cancer, breast cancer, uveal melanoma, and neuroblastoma. Specific mutations in PKCβ can lead to an increased growth rate and cell viability of solid cancer tumor cells. Reproduced with permission from ref. (Parker et al., 2021). Copyright 2020, Springer Nature.

PKC is a widely expressed family of serine/threonine kinases, with multiple isoforms, and is categorized into three functionally unique subgroups: conventional, novel and atypical (Baffi et al., 2019), and these categories are characterized by molecules that produce signal transduction^28.^ The conventional PKCs, PKCα, PKCβ and PKCγ (Lin and Takemoto, 2005; Breitkreutz et al., 2007), are activated by certain phospholipids, diacylglycerol, and calcium. The novel PKCs, which require calcium for activation, are PKCδ, PKCε, PKCθ, and PKCη (Lin and Takemoto, 2005; Breitkreutz et al., 2007). The remaining PKCs are activated independent of calcium and diacylglycerol, and the remaining PKCs are not targets for the current PKC inhibitors (Pfeifhofer et al., 2006). As shown in Figure 4, the D427 and R471/474 mutations in the kinase domains of PKCα and PKCβ facilitate tumor growth (Pears et al., 1990; Silva-Rodríguez et al., 2022). The inhibition of PKCδ and PLCβ significantly inhibits uveal melanoma cell proliferation and decreases the size of melanoma cells (Thuille et al., 2019). Following the activation of PKC, the RAS-dependent extracellular signal-regulated kinase (ERK1/2) pathway activates rapidly accelerated fibrosarcoma (RAF)/MAPK, a pathogenic pathway that allows GNAQ-mutated uveal melanoma to progress and eventually become metastatic (He et al., 2014).

### Darovasertib: a novel agent targets GNAQ/GNA11 mutations and PKC pathway

**FIGURE 5:**
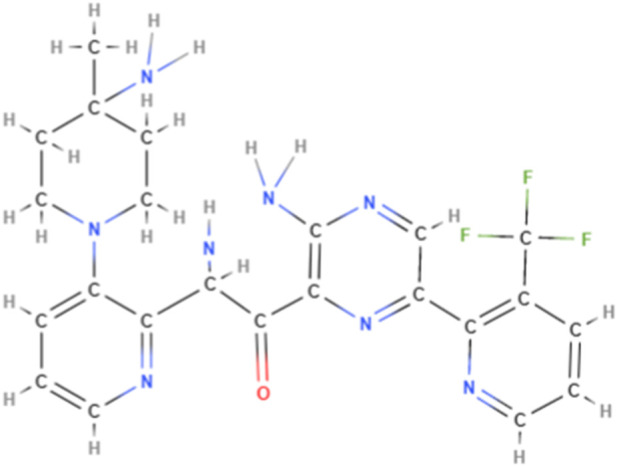
The chemical structure of darovasertib. The molecular weight of darovasertib is 472.48.

Darovasertib (3-Amino-N-[3-(amino-4-methylpiperdin-1-yl)pyridine-2-yl]-6-[3-(trifluoromethyl)pyridine-2-yl]pyrazine-2-carboxamide; also known as LXS196, as shown in Figure 5), a novel PKC inhibitor that targets uveal melanoma with GNAQ and GNA11 mutations, has been evaluated in a Phase I clinical trial and is currently being evaluated in a Phase I/II trial (Wei et al., 2022). Darovasertib potently inhibits the activity of the novel (δ, ϵ, η, θ) and classical (α, β) PKC isoforms, which inhibits the PKC signaling pathway (Wei et al., 2022). It also inhibits the proliferation of uveal melanoma and significantly decreases cell viability in metastatic uveal melanoma (Wei et al., 2022).

## Preclinical results

Hepatocyte stimulation factor, HGF, is present in the tumor microenvironment of uveal melanoma (Naldini et al., 1991) and recent studies have reported that a high HGF level is significantly correlated with metastatic uveal melanoma (Tanaka et al., 2021). The expression of HGF is upregulated by the activation of the MAPK and PI3K pathways (Tanaka et al., 2021). HGF is the endogenous cognate ligand for the tyrosine kinase, c-mesenchymal to epithelium transition protein (cMET) (Tanaka et al., 2021). HGF induces uveal melanoma cell proliferation and survival, which produces resistance to MET inhibitors, such as crizotinib and cabozantinib, in metastatic uveal melanoma tumors (Croce et al., 2019). A recent *in vitro* study indicated that HGF promotes resistance to MEK inhibitors by increasing the expression of the proteins, Bcl-2-like 11 (BIM)—extra-long (EL) and Bcl-2 Modifying Factor (BMF), in uveal melanoma cells (Schoumacher et al., 2016). The selective inhibition of the catalytic activity of the PI3K/AKR mouse strain thymoma (AKT) pathway may decrease the incidence of resistance to MEK inhibitors in metastatic uveal melanoma (Frey et al., 2020). Thus, it has been hypothesized that a combination of PKC and MEK inhibitors may be an effective treatment for uveal melanoma tumors resistant to the MET inhibitor, crizotinib (Croce et al., 2019).

Based on preclinical data indicating that the activation of parallel signaling pathways facilitates cell proliferation, despite the inhibition of MEK1/2, studies were subsequently conducted to determine whether the combination of darovasertib, an inhibitor of the novel (δ, ϵ, η, θ) and classical (α, β) PKC isoforms and crizotinib, a MET inhibitor, would be efficacious in patients with metastatic uveal melanoma tumors resistant to MET inhibitors (Croce et al., 2019). In uveal melanoma tumors, the PI3K/AKT and PKC/MAPK pathways are highly activated, suggesting a rationale for inhibiting these signaling cascades (Croce et al., 2019), and one *in vitro* study determined the effect of hepatocyte stimulation factor (HGF) in uveal melanoma cell lines (Tanaka et al., 2021). The metastatic uveal melanoma cell lines, MEL-202 (primary) and MM28 (metastatic) were incubated with darovasertib (2, 4, 6, 8, or 10 μM) and HGF (20, 40, 60, 80, or 100 ng/mL or crizotinib). The results indicated that: 1) in the absence of HGF, darovasertib inhibited the PKC proteins, phosphorylated Myristoylated alanine-rich C-kinase substrate (pMARCKS), phosphorylated Extracellular signal-regulated kinase (pERK) and PKCδ; 2) the addition of HGF significantly antagonized the inhibitory effect of darovasertib on pMARCKS, pERK and PKCδ, at a concentration ≥1.23 ng/mL. The decrease in the efficacy of darovasertib in the cell lines could have been due to a low level of c-MET receptors and 3) darovasertib, at 80 nM (a plasma concentration found in humans treated with darovasertib that is safe and tolerable), produced a significant synergistic efficacy, in combination with 80 nM of crizotinib, in the MEL202 and MM28 cell lines (Frey et al., 2020). This effect was likely due to the darovasetib and crizotinib inhibiting the activation of the PKC and MET pathways, respectively. These data suggest that the MAPK and PI3K pathways may be activated by high levels of HGF, which decreases the efficacy of darovasertib, and this can be overcome by incubating the two uveal melanoma cell lines with crizotinib, a MET inhibitor (Camidge et al., 2014).

In the preclinical and clinical studies related to metastatic uveal melanoma, the accurate measurement of the response to therapy is a problem (Kaliki et al., 2015). Furthermore, the prognosis for metastatic uveal melanoma is poor (Kaliki et al., 2015). Given that more than 95% of uveal melanoma tumors contain activating driver mutations that can be identified in the blood, the levels of circulating tumor DNA (ctDNA) levels have been hypothesized to be an indicator of the therapeutic response, as ctDNA levels are positively correlated correlate with the tumor burden, thereby representing an excellent prognostic biomarker (Bidard et al., 2014). In a study by Park et al. (Park et al., 2021), plasma samples were collected from 17 metastatic uveal melanoma patients who had been treated with darovasertib in a Phase I clinical trial, and ctDNA levels were determined using the Next-Generation Sequencing (NGS) method. The results indicated that the baseline level of ctDNA was positively correlated with the baseline tumor burden and the level of the biomarker, lactate dehydrogenase (LDH). There was no significant difference in the baseline ctDNA levels among the patients treated with darovasertib after they were recruited into the clinical trial. The ctDNA levels collected were significantly positively correlated to the patient’s therapeutic response to darovasertib. Patients that benefited from treatment with darovasertib had significantly lower ctDNA levels 14–30 days after the initiation of therapy, compared to patients that did not achieve a clinical benefit. Mutations in p53, a protein that is commonly mutated in many types of cancer (Park et al., 2021), were also detected in ctDNA using NGS (Kapiteijn et al., 2019).

## Past and current clinical trials conducted with darovasertib

As shown in Table 2, darovasertib has been evaluated in a Phase I trial. The first Phase I clinical trial with darovasertib evaluated its safety, efficacy, pharmacodynamic and pharmacokinetic profile (Kapiteijn et al., 2019). The trial enrolled 68 patients, who received LXS196 (Darovasertib) at doses ranging from 100 to 1,000 mg once daily (38 patients) and 200–400 mg twice daily (30 patients). The first 38 patients received treatment once a day but due to toxicity at doses ≥500 mg, treatment was given twice a day (200–400 mg). The recommended dose escalation (RDE) was 300 mg twice daily. All patients who received treatment once a day discontinued the trial because of progressive disease and intolerable adverse effects. Five out of 30 patients that were given treatment twice daily remained in the trial. Of the five remaining patients receiving treatment twice daily, two maintained a partial response (at 200 and 300 mg BID), and 3 had an incomplete response or stable disease (300 mg BID). All five patients remained in the clinical trial for >13 months. The median progression-free survival was 4.14 months (95% CI 3.52–9 months) and the median duration of stable disease was 5.37 months in patients treated twice daily. Unlike AEB071 (Sotrastaurin), which from 450–1,100 mg, either BID or TID, did not significantly decrease the levels of the biomarkers, pMARCKS and pPKCδ (Kovarik and Slade, 2010), darovasertib (200 mg–400 mg twice daily) significantly decreased the levels of pMARCKS and pPKCδ (Wagle et al., 2020).

**TABLE 2:** The results of clinical trials of darovasertib.

	Intervention	Indication	Trial type	Number	Efficacy	Adverse effects	Trial number	Status
1	Group 1: darovasertib as a single agent Group 2: darovasertib and HDM201	metastatic uveal melanoma (MUM)	Phase I	107	2/17 (12%) had confirmed PR and 12/17 (71%) had stable disease as their best response PKPD: Rapidly absorbed, with a Tmax of ∼1 h post-dose, and a terminal half-life of 11 h for all doses	hypotension (22.1%) nausea (66.2%), diarrhea (45.6%), vomiting (30.9%), elevated ALT (22.1%), fatigue (20.6%). Grade 3 and 4 adverse effects occurred in 17 patients (25.0%)	NCT02601378/CLXS196X2101	Finished
2	Group 1: darovasertib monotherapy Group 2: Dose Escalation binimetinib+ darovasertib Group 3: Dose Escalation Crizotinib Combination	solid tumors harboring GNAQ and GNAQ/11 mutations or PRKC fusions (including MUM)	Phase Ib/II	254	Preliminary data: Group 1: 1-year (OS) rate: 57% median OS: 13.2 months decrease in tumor size in 46/75 MUM (61%) Group 2: 79% reported decrease in tumor size Group 3: 4/13 (31%): confirmed (PR); 46% (6/13) had tumor size decrease	No results	NCT03947385	Ongoing
3	Darovasertib monotherapy	primary uveal melanoma	Phase II	NA	No results	No results	NCT05907954	recruiting

The most frequent adverse effects (all grades, involving ≥20% of patients) reported in patients treated with darovasertib were hypotension (22.1%) nausea (66.2%), diarrhea (45.6%), vomiting (30.9%), increased levels of alanine transaminase (ALT, 22.1%) and fatigue (20.6%). The majority of grade 3 and 4 adverse effects occurred in 17 patients (25.0%) and hypotension was the most frequent. The twice-daily dosing schedule was safer than the QD dosing schedule, as patients treated twice daily reported fewer grade 3 or 4 adverse effects (20% for twice-daily treatment vs 28.9% with QD dosing) and fewer drug-related adverse effects (6.7% for twice-daily treatment vs 15.8% with QD). The most common adverse effects due to darovasertib (any grade involving >15% of patients), at the real dose escalation (n = 18), were nausea (77.8%), diarrhea (61.1%), vomiting (38.9%), liver impairment and increased ALT levels (27.8%), asthenia, dry skin, and rash (22.2%), hypotension, fatigue, increased aspartate aminotransferase (AST), dermatitis acneiform and peripheral edema (16.7%).

Pharmacokinetic data indicated that darovasertib was rapidly absorbed, with a T_max_ of ∼1 h post-dose, and a terminal half-life of 11 h for all doses. Doses of 300 mg once a day or 200 mg twice a day are assumed to be efficacious, based on the preclinical results, while the actual efficacious range will be determined based on an ongoing Phase I/II trial.

Based on the efficacy and safety data, a multi-center, open-label phase I/II trial will be conducted to determine the efficacy and safety of darovasertib in patients with solid tumors that contain either PRKC (Wagle et al., 2020) (a gene that codes for a transmembrane Ser/Thr kinase) fusions (i.e., a gene fuses with other unrelated genes) or GNAQ/GNA11 mutations. The trial plans to enroll 254 patients and the majority of these patients will most likely have metastatic uveal melanoma. Patients will be randomly divided into seven different cohorts that will receive either: 1) darovasertib monotherapy or 2) darovasertib in combination with binimetinib or darovasertib in combination with crizotinib (ClinicalTrials.gov Identifier: NCT03947385) (IDEAYA, 2021).

The preliminary results in patients treated with darovasertib monotherapy indicated that:

1) The one-year-overall survival (OS) rate was 57% (95% CI of 44%, 69%) in the second line, third line, and heavily pre-treated metastatic uveal melanoma patients. Compared to the 1-year overall survival rate in similar patients treated with AEB071 (37%), these data indicated that darovasertib monotherapy significantly increases the overall survival. Among these metastatic uveal melanoma patients, the median overall survival was 13.2 months, and the median overall survival was significantly greater than the historical median overall survival in similar populations, which was approximately 7 months.
2) There was a decrease in tumor size in 46 of the 75 metastatic uveal melanoma patients (61%) and 15 patients (20%) had an ideal therapeutic outcome, i.e., >30% decrease in the target lesion. One patient had a confirmed complete response. In the skin melanoma cohort, 80% (n = 4) of the evaluated patients (n = 5) had a decrease in tumor size and one patient had a confirmed partial response.

Preliminary results for patients treated with darovasertib and binimetinib (IDEAYA Biosciences, 2021) indicated that:

1) Two partial responses occurred out of nine metastatic uveal melanoma patients, based on the results of last two post-baseline scans (22%). One patient had a confirmed partial response and another patient had an unconfirmed partial response (−40.5%).
2) Among the evaluated metastatic uveal melanoma patients, 79% had a decrease in tumor size, based on at least one post-baseline scan and there were two partial responses (1 confirmed, one pending confirmatory scans) out of 9 patients that had at least two post-baseline scans.

Finally, the phase II trial results indicated that the combination of darovasertib (200 mg twice a day) with crizotinib (250 mg orally twice daily) produced a synergistic decrease in tumor size in patients with metastatic uveal melanoma. (ClinicalTrials.gov Identifier: NCT03947385) (Wagle et al., 2021). The results of this clinical trial indicated that:

1) Among the 16 patients evaluated, 100% of the patients had >1 post-baseline scan that showed a decrease in tumor size and a delay in tumor progression.
2) 4 of 13 (31%) patients had a confirmed partial response (PR), based on > 2 post-baseline scans, and no patients discontinued treatment before the second scan.
3) 46% of patients (6 of 13) had a >30% decrease in tumor size, based on > 2 post-baseline scans and one patient had an unconfirmed partial response.
4) No grade 4 or 5 adverse events occurred.

## Discussion

Although darovasertib has been reported to be efficacious in Phase I and II clinical in patients with uveal melanoma, Phase III trials must be conducted to provide additional data regarding its efficacy.

A recent study reported that PKC inhibitor monotherapy cannot suppress multiple active pathways in uveal melanoma tumors. The incubation of 11 different uveal melanoma cell lines (92.1, MP46, Mel270, MP38, OMM1.3, OMM1.5, MP41, Mel285, Mel290, Mel202) with darovasertib, 1 or 5 μM, produced cell cycle inhibition but not cell death in the majority of the GNAQ/GNA11-mutant cell lines (Park et al., 2022). Furthermore, darovasertib only significantly decreased the viability in the uveal melanoma cell lines, Mel270 and OMM1. Thus, although the inhibition of PKC activity by darovasertib significantly inhibits MAPK activity, it did not induce cell death in the majority of uveal melanoma, as PKC inhibition does not inhibit multiple activated Gα pathways downstream of PKC (Robertson et al., 2017). This may be due to the positive correlation of MAPK activity with uveal melanoma cell proliferation but not cell survival (Park et al., 2022). Consequently, the PKC inhibitors decrease cell proliferation but not cell death. It has been reported that c-MET is expressed on uveal melanoma cells (Barisione et al., 2015) and the combination of c-MET inhibitors like crizotinib with PKC inhibitors may be useful in treating patients with uveal melanoma (Hitchman et al., 2021).

Currently, other drugs are being developed and evaluated for the treatment of metastatic uveal melanoma. AEB071 (i.e., sotrastaurin), an inhibitor of the PKC isoforms, PKC-α, PKC-β and PKC-δ.

The inhibition of PKC-β and PKC-δ (Wu et al., 2012a) by sotrastaurin was evaluated in a Phase I, single-arm, open-label trial for the treatment of metastatic uveal melanoma (Piperno-Neumann et al., 2020). The trial recruited 153 patients and the patients were given sotrastaurin either twice or three times daily, at doses ranging from 450 to 1,400 mg/day. Seventy-four percent of the patients discontinue treatment due to disease progression and 13% of patients discontinued treatment due to adverse effects. Ninety-seven percent of the patients treated with sotrastaurin experienced adverse effects, which was significantly greater than that for patients treated with darovasertib. The most common adverse effects were nausea (81%), dysgeusia (60%), constipation (58%), vomiting (58%), diarrhea (44%), chromaturia (39%), fatigue (32%), decreased appetite (31%) and asthenia (30%). Therapeutically, 4 out of 156 patients (3%) had a partial response, 76 out of 156 patients (50%) had stable disease and 34 (22%) patients had a ≥10% decrease in tumor size. The median progression-free survival was 3.5 months (95% CI, 2.5–3.6 months) (Musi et al., 2014).

The combination of sotrastaurin with the MEK inhibitor, binimetinib, has been reported to produce a synergistic effect in a xenograft mouse model (Sagoo et al., 2014). Nude mice were implanted with the GNAQ/GNA11 mutated human uveal melanoma cell line, 92-1, and the tumors were allowed to grow for 12 days. The combination treatment was given 21 days (Chen et al., 2014). The intravenous administration of 40 mg/kg, three times daily of sotrastaurin and 3.5 mg/kg, twice daily of binimetinib, produced a 12% decrease in tumor size, compared to mice treated with placebo. The intravenous administration of 80 mg/kg orally of sotrastaurin, three times daily and 3.5 mg/kg orally of binimetinib, twice daily, produce a 52% decrease in tumor size and volume. Based on these results, a Phase Ib/IIa clinical trial (NCT01801358) was conducted to determine the efficacy and safety of sotrastuarin and binimetinib in patients with metastatic uveal melanoma. However, the FDA website indicated that the trial was terminated early because of poor tolerability and limited efficacy, as half of the patients (19/38) reported severe drug-related adverse events and the median PFS was only 3.1–4 weeks (Steeb et al., 2018).

Another PKC inhibitor, LY317615 (enzastaurin), is a potent and competitive inhibitor of PKCβ at low concentrations (0.006 μmol/l) and other isoforms at higher concentrations (PKCα = 0.039 μmol/L, PKCγ = 0.083 μmol/L and PKCε = 0.110 μmol/L) and it targets, PI3K/AKT, Glycogen Synthase Kinase 3 Beta (GSK3 β) and ribosomal protein S6^63^. Enzastaurin significantly decreased the phosphorylation of glycogen synthase kinase 3β and the phosphorylation of ribosomal protein S6 and AKT, which decreased the activation of the PKCβ and AKT pathways (Ma and Rosen, 2007). *In vitro* data indicated that enzastaurin produced a significant antiproliferative and pro-apoptotic effect, as indicated by a decrease in the survival and viability of cell lines with GNAQ/GNA11 mutations (Wu et al., 2012c).

Overall, compared to other PKC inhibitors, darovasertib produced a greater inhibition of the PKC proteins, novel (δ, ϵ, η, θ) and classical (α, β) isoforms, and downstream signaling pathways, and a lower rate of grade III and IV adverse events. A phase III clinical trial, evaluating the efficacy of darovasertib in patients with metastatic uveal melanoma, will be initiated upon the completion of an ongoing Phase I/II trial.

## Future directions

As a result of the efficacy of darovasertib in patients with metastatic uveal melanoma, a Phase II clinical trial will be conducted to determine if darovasertib is safe, tolerable, and efficacious as neoadjuvant/adjuvant therapy in patients with ocular melanoma (ClinicalTrials.gov Identifier: NCT05187884). The estimated enrollment is 12 patients and eligible patients will receive up to 4 weeks of treatment with darovasertib (300 mg, BID) (Clinicaltrials, 2023). Patients that have an initial response will undergo adjuvant therapy with darovasertib (300 mg, BID) for 6 months. The primary outcome will be the evaluation of the safety of a 4 week treatment course, using the Common Terminology Criteria for Adverse Events (CTCAE) v5.0 guidelines and the percentage of participants that complete the 4 week treatment period. The secondary outcomes to be determined are the 1) therapeutic effect of neo-adjuvant darovasertib on the decrease in uveal melanoma tumor size and 2) time to recurrence/disease-specific survival in patients treated with adjuvant darovasertib, using the Response Evaluation Criteria In Solid Tumors (RECIST) 1.1, the standard for determining the effect of a treatment on tumor size using imaging techniques (Schwartz et al., 2016). The trial was first updated on the FDA website clinicaltrial.gov. on 12 January 2022 and it is still in the recruiting phase (Ideayabio, 2022b).

There are *in vitro* and *in vivo* studies being conducted with darovasertib to determine if it can be used in combination with the KRAS inhibitors, sotorasib and adagrasib, to treat non-small cell lung cancer and hepatocellular carcinoma (Reck et al., 2021). The results of these ongoing studies remain to be published.

Data suggests that darovasertib may be used to treat diseases other than melanoma and solid tumors. A recent *in vitro* study was conducted to determine if darovasertib would have efficacy in treating cerebral ischemia (Wang et al., 2022). Numerous studies have shown that the excessive accumulation of glutamate plays a role in producing brain damage that occurs during the early stages of cerebral ischemia (Zhang et al., 2019). Glutamate transporter-1 (GLT-1), a sodium-dependent transporter, expressed mainly by astrocytes and axonal nerve terminals in the brain, mediates glutamate homeostasis by removing excess glutamate (Wang et al., 2022). An *in vivo* study in mice reported the intravenous administration of 6 mg/kg of darovasertib significantly decreased the expression of GLT-1 in the hippocampus, which decreased glutamate levels by 33% (Wang et al., 2022). Thus, darovasertib could be a potential treatment for cerebral stroke and cerebral ischemia, although this remains to be determined.

Clinical trials have indicated limitations for the use of darovasertib. Since darovasertib decreases uveal melanoma cell proliferation but does not directly produce cell death, it is more efficacious when used in combination with other drug regimens, and the efficacy of the combination also depends on what other medication is used in the combination. Thus, to optimize the uveal melanoma therapy and obtain greater efficacy in the future, studies could be conducted with darovasertib, in combination with other drugs that inhibit other tyrosine kinases, such as VEGF-B and PD-1/CTLA-4 inhibitors.

## Conclusion and perspectives

Uveal melanoma has a high risk of progressing to metastatic uveal melanoma, despite patients receiving current standard treatments, such as brachytherapy, enucleation, and external beam radiotherapy (Weis et al., 2016). The high frequency of GNAQ/GNA11 mutations in metastatic uveal melanoma makes them an ideal target for novel therapies. Recent studies have shown that inhibiting the PKC pathway can downregulate the activation of the MAPK pathway mediated by GNAQ/GNA11 mutations. The novel drug, darovasertib, is a first-in-class drug that inhibits novel (δ, ϵ, η, θ) and classical (α, β) isoforms of PKC. The use of darvoasertib, in combination with binimetinib (a MEK inhibitor), significantly decreased the size of metastatic uveal melanoma tumors. Furthermore, darovasertib is also more tolerable than AEB071 (Wu et al., 2012b), a compound that inhibits PKC α, β, and δ, in metastatic uveal melanoma tumors. Future clinical trials (NCT05187884) will be conducted to determine if darovasertib can be used as adjuvant/neoadjuvant therapy in ocular melanoma (Clinicaltrials, 2023). Finally, recent *in vitro and in vivo* data suggest darovasertib may represent a novel treatment for cerebral ischemia (Wang et al., 2021), although this remains to be determined in clinical trials.

## Abbreviations

ERK1/2, Ras-dependent extracellular signal-regulated kinase; GNAQ, G protein subunit alpha q HGNC ID: HGNC:4390; GNA11, G protein subunit alpha 11 HGNC ID: HGNC:4379; HGF, Hepatocyte stimulation factor; IGF-1, insulin-like growth factor-1; IGF-1R, insulin-like growth factor type 1 receptor; MEK inhibitors, Mitogen-activated protein kinase/Extracellular inhibitors; MAPK, Mitogen-activated protein kinases; PI3K, phosphoinositide-3-kinase; PKC inhibitors, Protein kinase C inhibitors; UV, uveal melanoma; YAP, Hippo-Yes-associated protein 1.
